# Fast myocardial perfusion SPECT denoising using an attention-guided generative adversarial network

**DOI:** 10.3389/fmed.2023.1083413

**Published:** 2023-02-03

**Authors:** Jingzhang Sun, Bang-Hung Yang, Chien-Ying Li, Yu Du, Yi-Hwa Liu, Tung-Hsin Wu, Greta S. P. Mok

**Affiliations:** ^1^Biomedical Imaging Laboratory (BIG), Department of Electrical and Computer Engineering, Faculty of Science and Technology, University of Macau, Taipa, Macao SAR, China; ^2^Department of Biomedical Imaging and Radiological Sciences, National Yang Ming Chiao Tung University, Hsinchu, Taiwan; ^3^Department of Nuclear Medicine, Taipei Veterans General Hospital, Taipei City, Taiwan; ^4^Department of Internal Medicine, Yale University School of Medicine, New Haven, CT, United States; ^5^Center for Cognitive and Brain Sciences, Institute of Collaborative Innovation, University of Macau, Taipa, Macao SAR, China; ^6^Ministry of Education Frontiers Science Center for Precision Oncology, Faculty of Health Science, University of Macau, Taipa, Macao SAR, China

**Keywords:** denoising, attention-guided, deep learning, myocardial perfusion, fast SPECT

## Abstract

**Purpose:**

Deep learning-based denoising is promising for myocardial perfusion (MP) SPECT. However, conventional convolutional neural network (CNN)-based methods use fixed-sized convolutional kernels to convolute one region within the receptive field at a time, which would be ineffective for learning the feature dependencies across large regions. The attention mechanism (Att) is able to learn the relationships between the local receptive field and other voxels in the image. In this study, we propose a 3D attention-guided generative adversarial network (AttGAN) for denoising fast MP-SPECT images.

**Methods:**

Fifty patients who underwent 1184 MBq ^99m^Tc-sestamibi stress SPECT/CT scan were retrospectively recruited. Sixty projections were acquired over 180° and the acquisition time was 10 s/view for the full time (FT) mode. Fast MP-SPECT projection images (1 s to 7 s) were generated from the FT list mode data. We further incorporated binary patient defect information (0 = without defect, 1 = with defect) into AttGAN (AttGAN-def). AttGAN, AttGAN-def, cGAN, and Unet were implemented using Tensorflow with the Adam optimizer running up to 400 epochs. FT and fast MP-SPECT projection pairs of 35 patients were used for training the networks for each acquisition time, while 5 and 10 patients were applied for validation and testing. Five-fold cross-validation was performed and data for all 50 patients were tested. Voxel-based error indices, joint histogram, linear regression, and perfusion defect size (PDS) were analyzed.

**Results:**

All quantitative indices of AttGAN-based networks are superior to cGAN and Unet on all acquisition time images. AttGAN-def further improves AttGAN performance. The mean absolute error of PDS by AttcGAN-def was 1.60 on acquisition time of 1 s/prj, as compared to 2.36, 2.76, and 3.02 by AttGAN, cGAN, and Unet.

**Conclusion:**

Denoising based on AttGAN is superior to conventional CNN-based networks for MP-SPECT.

## 1. Introduction

Myocardial perfusion single photon emission computed tomography (MP-SPECT) is a standard method for the quantitative diagnosis of coronary artery disease (CAD) (1). However, the acquisition time for the conventional NaI-based MP-SPECT is relatively long (15-20 min) (2), leading to potential motion artifacts, e.g., upward creep (3), patient’s discomfort, lower patient throughput (4), and mismatch artifacts between the sequential MP-SPECT and CT (5, 6). New scanner geometries with parallel-hole (7) or multi-pinhole collimations (8, 9) for MP-SPECT are proposed for better photons detection efficiency and reduced scan time (2-8 min) (10, 11). Advanced reconstruction algorithms (12) also facilitate the possibility of reducing acquisition time without degrading image quality. However, the acquisition time for MP-SPECT is still much longer than CT in general (4). Therefore, it is necessary to pursue fast MP-SPECT, without compromising the image quality and diagnostic accuracy.

Image noise is a substantial problem for fast MP-SPECT due to the limited detected counts and the fact that it degrades the image quality, hampering clinical diagnosis and quantification results (9). Recently, deep learning (DL) methods are promising to reduce the noise for MP-SPECT images. Ramon et al. (13) proposed 3D convolutional neural networks (CNN) to denoise the reconstructed MP-SPECT images with reduced injected dose. Liu et al. used a 3D Unet trained on a noise-to-noise strategy for denoising full dose MP-SPECT reconstructed images and showed improved results as compared to the use of traditional filter (14). They further evaluated the performance of DL-based denoising according to the area under the curve (AUC) of the total perfusion deficit (TPD) scores results (15). Aghakhan et al. (16) used a 2D conditional generative adversarial network (cGAN) for denoising the reduced dose MP-SPECT images from 1/8 to 1/2 dose levels in the projection domain. Shiri et al. (17) proposed a 2D residual CNN (ResNet) to estimate full time (FT) MP-SPECT projection images. Previously, our group implemented a 3D cGAN to denoise fast and low dose MP-SPECT reconstruction (18) and projection (19) images. Our results showed that denoising on the projection domain is superior to the reconstruction domain (19).

However, conventional CNN-based methods use fixed-sized convolutional kernels to convolute one local region within the receptive field at a time, which would be ineffective for learning the feature dependencies across large regions (20). The feature dependencies across large regions can only be learned when the feature maps are down-sampled into a relatively small matrix size after passing through several convolutional layers (21). The attention mechanism has shown to be effective in capturing the long-range dependencies of structural information across large regions (20). It has been implemented for CT segmentation (22) and low dose CT denoising (23). In this study, we propose an attention-based cGAN (AttGAN) in denoising fast MP-SPECT projection images and compare its performance with Unet-based and cGAN-based denoising. We further incorporate the patient defect information into the network to improve the AttGAN performance.

## 2. Materials and methods

### 2.1. Patient dataset

Fifty anonymized patients who underwent routine stress SPECT/CT scan ∼30 minutes post ^99m^Tc-sestamibi injection on a clinical SPECT/CT system (NM/CT 870 CZT, GE Healthcare, USA) were retrospectively enrolled in this study under local ethics approval (IRB number 2022-11-002CC, Table 1). Among them, 18 were read as having at least a cardiac defect, which had perfusion abnormalities, according to their medical records from SPECT images and clinical histories. Before the SPECT acquisition, a helical CT scan (120 kVp, smart mA (10-150 mA), 0.375 cm slice thickness) was acquired in the heart region for attenuation correction in SPECT reconstruction. The CT reconstruction matrix size was 512 × 512 × variable axial coverage, with a voxel size of 0.9765 mm. Patients were injected with 1,184 MBq ^99m^Tc-sestamibi, and 60 projections were acquired through 180° from the right anterior oblique to the left posterior oblique positions with a matrix size of 64 × 64. The primary photopeak energy window was centered at 140.5 keV with a 20% width and the scatter window was centered at 120 keV with a 10% width.

**TABLE 1:** Demographic information for the patient study.

	Female	Male	Total
Gender	13 (26%)	37 (74%)	50 (100%)
Age (years)	69.2 ± 9.73 (56-90)	64.9 ± 11.24 (42-83)	66.0 ± 10.94 (42-90)
BMI (kg/m^2^)	24.5 ± 3.13 (21.09-30.47)	25.0 ± 2.65 (17.91-30.11)	25.0 ± 2.92 (17.92-31.60)
Perfusion defect size (PDS, %)	3.23 ± 2.20 (0-7)	4.46 ± 5.57 (0-29)	4.14 ± 4.92 (0-29)
Cardiac defect	2 (4%)	16 (32%)	18 (36%)
**CAD risk factors**			
Hypertension	6 (12%)	20 (40%)	26 (52%)
Dyslipidaemia	8 (16%)	21 (42%)	29 (58%)
Diabetes	2 (4%)	14 (28%)	16 (32%)
Smoker	0 (0%)	8 (16%)	8 (16%)
Family history of CAD	4 (8%)	9 (18%)	13 (26%)

Mean ± SD and range are presented for age, BMI, and PDS.

The original acquisition time was 10 s/view. We also obtained various fast MP-SPECT projection images by reducing the projection acquisition time to be 7, 5, 3, 2, and 1 s based on the list mode data of the FT images, respectively. All clinical data were reconstructed by the 3D ordered subset expectation maximization (OS-EM) algorithm with 5 iterations and 4 subsets, with CT-based attenuation and dual energy window scatter corrections. The reconstruction matrix size was 64 × 64 × 19 with a voxel size of 0.6096 cm. A 3D post-reconstruction Gaussian filter with a standard deviation of 0.6 voxel was applied on the FT images for data analysis.

### 2.2. Attention-guided generative adversarial network (AttGAN)

**FIGURE 1:**
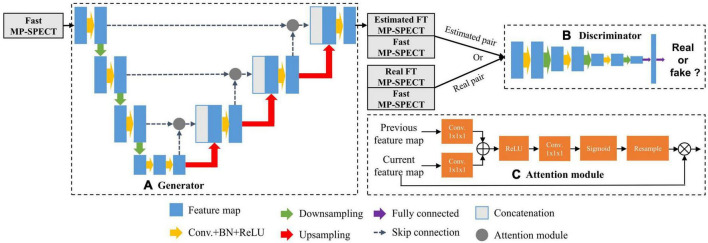
The AttGAN architecture used in this study. **(A)** Generator; **(B)** discriminator; **(C)** attention module.

The architecture of the AttGAN used in this study is shown in Figure 1 (20). Similar to cGAN, AttGAN is comprised of two subnetworks: a generator (Figure 1A) and a discriminator (Figure 1B). The generator, which was conditioned with fast MP-SPECT projection images, transformed the fast MP-SPECT images into estimated FT MP-SPECT projection images. The estimated images were later paired with the original fast MP-SPECT projection images as an estimated sample pair. The fast MP-SPECT projection images were also paired with the corresponding FT MP-SPECT projection images as a real sample pair. The discriminator learned to differentiate between the estimated sample pairs and the real sample pairs.

The Unet-based generator (24) had subunits of encoding, bottleneck, and decoding layers. Each encoding layer was comprised of convolution (3 × 3 × 3), batch normalization (BN) (25), rectified linear unit (ReLU) activation, and dropout with a rate of 50%, followed by max-pooling to down-sample feature maps between layers. The decoding layers mirrored the encoding layers, except the up-sample layers replaced the down-sample layers, and skip connection between the encoding and decoding layers was added. The discriminator was a CNN-based network used in our previous study (19).

The attention modules, which were used for calculating the relationships of each voxel to all other pixels within a feature map, were incorporated together with the skip-connection between the encoding layers and decoding layers in the generator (Figure 1C) (20). The feature maps *x* from the previous encoding layer *g* and current decoding layer *f* were transformed by a convolution (1 × 1 × 1) respectively, where *g*(*x*) = *W*_*g*_*x* and *f*(*x*) = *W*_*f*_*x*. *W_g_* and *W_f_* were trainable parameters. The feature maps of decoding layer *f*(*x*) were then down-sampled to be consistent with the size of *g*(*x*). We performed an inner product of the two vectors *g*(*x*)and *f*(*x*) to obtain the feature dependencies between every two voxels:


$$\alpha_{\text{i},\text{j}}=\text{f}\,{\left({\text{x}}_{\text{i}}\right)}^{\text{T}}\cdot \text{g}\,\left({\text{x}}_{\text{j}}\right)$$

The α_i,j_ further went through ReLU activation, convolution (1 × 1 × 1), and softmax function to normalize and reshape the feature maps to become *r*_i,j_. Finally, we multiplied *r*_i,j_ with the feature maps *x* from the previous encoding layer *g* to obtain the attention coefficients *Att*:


$$\text{Att}=\sum_{\text{i}=1}^{\text{N}}{\text{x}}_{\text{i}}\cdot {\text{r}}_{\text{i},\text{j}}$$

The *L_1_* loss (26) and the adversarial loss *L*_*ADV*_ were used for training the generator *g*. The discriminator was trained by a cross-entropy loss *L_D_* (19). The final objective function of AttGAN was:


$$L_{\text{AttGAN}}={\text{argmin}}_{\text{G}}\,{\text{max}}_{\text{D}}\,\left(L_{\text{ADV}}\,\left(\text{G},\text{D}\right)+\lambda \,{\text{L}}_{1}\,\left(\text{G}\right)\right)$$

where λ is set to be 100 to adjust the weight of *V_L_*_1_ (*G*) (26). The AttGAN was trained by minimizing the loss. The Unet and cGAN structures were the same according to our previous studies (18, 19).

### 2.3. Data preprocessing

**FIGURE 2:**
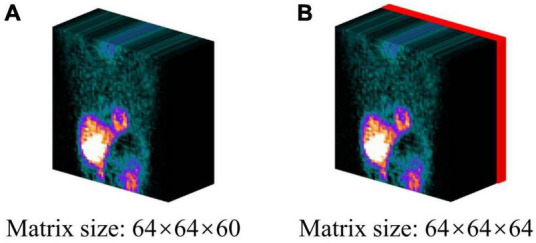
The projection datasets used in this study: **(A)** original projection, **(B)** projection incorporating the patient defect information. The defect information block (red) was encoded by binary values (0 = without defect/1 = with defect).

All the intensity values of MP-SPECT projection images were normalized to a range of 0–1 for training. In addition, we further incorporated the binary patient defect information, i.e., with (1) or without defect (0) from patients’ own medical records, by embedding four 64 × 64 slices with the same binary values into the projection images (Figure 2).

### 2.4. Network implementation

The AttGAN incorporating patient defect information (AttGAN-def), AttGAN, cGAN, and Unet were implemented using Tensorflow which ran on a NVIDIA GeForce RTX 2080Ti GPU. The Adam optimizer was applied to optimize this proposed model based on an initial learning rate of 0.0001 and trained to 400 epochs.

We performed a 5-fold cross-validation on the clinical datasets to evaluate four DL approaches for various fast SPECT acquisitions. Specifically, for each fold of evaluation, FT and fast SPECT projection images of 35, 5, and 10 patients were selected for training, validation, and testing, respectively. This process was repeated 5 times and all fifty patient datasets were tested and averaged for the final results. The denoised projections were further reconstructed using the same OS-EM algorithm with 5 iterations and 4 subsets with attenuation and scatter correction. No post-reconstruction filter was applied on the reconstructed images generated from the denoised projections for further analysis.

The hyper-parameters, e.g., number of layers and filters within each layer, were determined based on a training-validation procedure for AttGAN. Specifically, the number of layers varied as 2, 3, 4 and 5, while the number of filters within each layer varied as 8, 16, 24, 32 and 40. The hyper-parameters for cGAN and Unet were determined in our previous study (18, 19). The training time for AttGAN-def, AttGAN, cGAN, and Unet was 2.2, 2.2, 2.0, and 1.9 hr, respectively.

### 2.5. Data analysis

**FIGURE 3:**
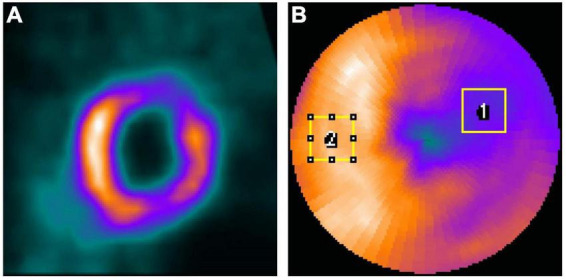
**(A)** The 3D VOI used for voxel-based error calculation. **(B)** Sample ROIs drawn from a polar plot for IR calculation on a selected patient. (ROI_1_ was for defect while ROI_2_ was for a uniform normal region).


The voxel-based error of the denoised images was assessed by the normalized mean square error (NMSE), structural similarity index (SSIM), peak signal-to-noise ratio (PSNR), joint histogram, and linear regression measured on a 3D volume-of-interest (VOI, 18 × 18 × 18, Figure 3A) which covered the whole heart. The filtered FT reconstructed MP-SPECT images were used as the reference.

NMSE=∑k=1N(ID-IFT)2∑k=1NIFT2

$$\text{SSIM}=\frac{\left(2\,\mu_{\text{D}}\,\mu_{\text{FT}}+{\text{C}}_{1}\right)\,\left(2\,\sigma_{\text{D},\text{FT}}+{\text{C}}_{2}\right)}{\left(\mu_{\text{D}}^{2}+\mu_{\text{FT}}^{2}+{\text{C}}_{1}\right)\,\left(\sigma_{\text{D}}^{2}+\sigma_{\text{FT}}^{2}+{\text{C}}_{2}\right)}$$

$$\text{PSNR}=10\cdot {\text{log}}_{10}\,\left(\frac{{\text{MAX}}_{\text{FT}}}{\sqrt{\text{MSE}}}\right)$$

where *I_D_* represents the voxel values in denoised reconstructed images, *I*_*FT*_ is the voxel values on the filtered FT reconstructed images, *N* (5832) is the number of voxels in the VOI, μ_*D*_ and μ_*FT*_ are the mean values of the denoised and reference images, σ_*D*_ and σ_*FT*_ are the standard deviations of the denoised and reference images respectively, and σ_*D*,*FT*_ is the cross-covariance between the two images. The constants *C_1_* and *C_2_* are set to be 0.01 and 0.02, respectively (17). *MAX*_*FT*_ indicates the maximum possible pixel value of the reference images while *MSE*indicates the mean squared error between the denoised and reference images.

Two regions-of-interest (ROI_1_ and ROI_2_) were drawn on the defect region and a uniform normal region on the polar plots based on visual assessment (Figure 3B) and were adjusted for each patient, respectively. The same ROIs were applied for all denoised images for the same patient. The intensity ratio (IR) was calculated from the mean value of the defect ROI (ROI_1_) divided by the mean value of the uniform ROI (ROI_2_). The absolute error of IR between FT images and different denoised images was computed.

A clinical relevant index, the perfusion defect size (PDS, %LV), i.e., an index similar to the total perfusion deficit, was measured by the Wackers-Liu CQ™ (WLCQ) software (Voxelon Inc, Watertown, CT) (27). The absolute error of PDS between FT and different denoised images was computed. The Bland–Altman plots were also computed to quantify the agreement of PDS. For the statistical analysis, a two-tailed paired t-test with Bonferroni correction (SPSS, IBM Corporation, Armonk, NY, USA) was performed between AttGAN-def and other denoising methods at different acquisition time/view for NMSE, PSNR, SSIM, PDS, and IR. A p-value of less than 0.05 was considered as statistically significant.

## 3. Results

### 3.1. Reconstructed images, polar plots, and 17-segment analysis

**FIGURE 4:**
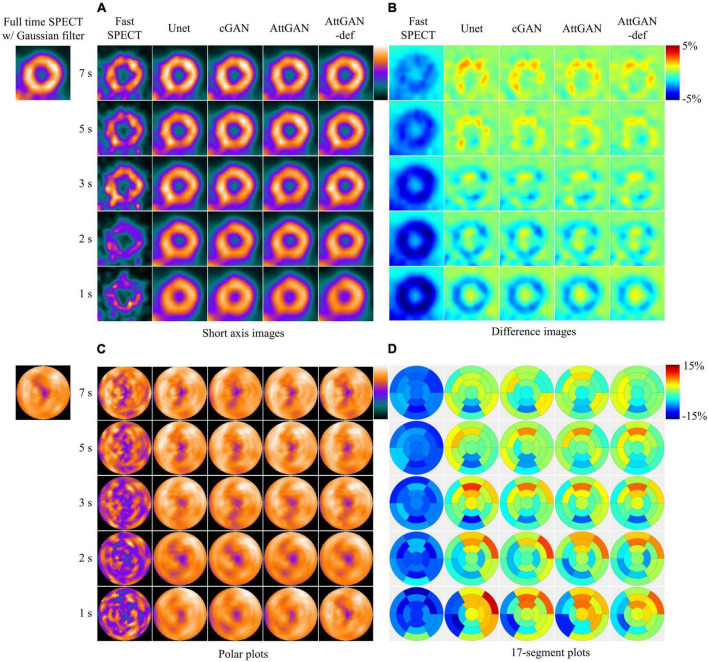
Sample images of a normal male patient (age = 67, BMI = 25.4) before and after DL-based denoising for five shorter acquisition times. The images are shown in **(A)** short axis images, **(B)** difference images as compared to FT SPECT, **(C)** polar plots, and **(D)** 17-segment plots.

**FIGURE 5:**
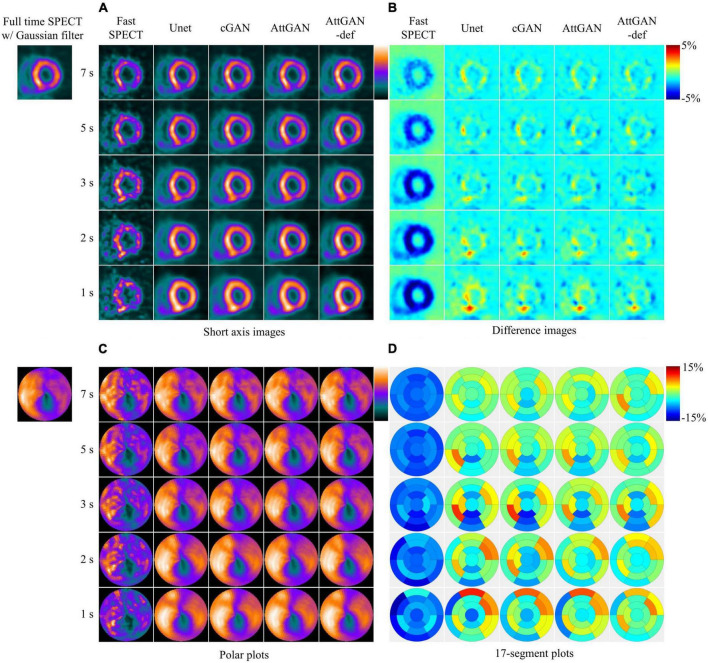
Sample images of another male patient (age = 77, BMI = 24.2) with an abnormal perfusion in the LAD and LCX territory before and after DL-based denoising for five shorter acquisition times. The images are shown in **(A)** short axis images, **(B)** difference images, **(C)** polar plots, and **(D)** 17-segment plots.

Figure 4 shows the short axis fast MP-SPECT images, their corresponding difference images as compared with filtered FT SPECT images, polar plots as well as 17-segment plots processed using different DL denoising methods for a normal male patient. Figure 5 shows the same results for a male patient with a defect in the left anterior descending (LAD) and left circumflex artery (LCX) region. It can be observed that all the DL-denoised fast SPECT images are similar to the filtered FT SPECT images based on a visual assessment, with the noise level notably suppressed. Furthermore, it is noted that the proposed AttGAN methods have less bias than Unet and cGAN methods according to their corresponding images. Less bias is also observed from the 17-segment images for the AttGAN and AttGAN-def methods. The denoised images consistently exhibit worse resolution, i.e., more blurring, in shorter acquisition times.

### 3.2. Quantitative analysis

#### 3.2.1. Physical and clinical indices

**FIGURE 6:**
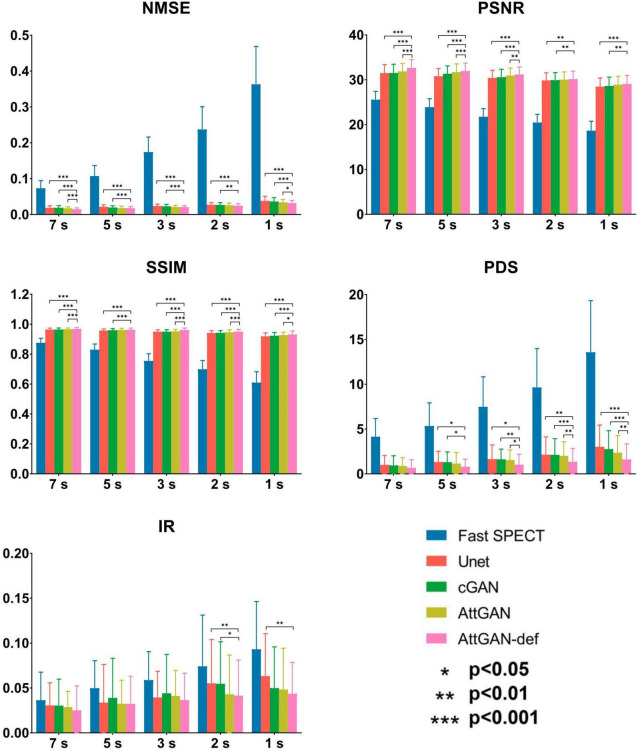
Quantitative comparison of NMSE, PSNR, SSIM, PDS, and IR on 50 testing datasets for fast SPECT and different denoised images for five shorter acquisition times. Error bars indicate the standard deviation. The filtered FT SPECT images were used as reference. *p<0.05, **p<0.01, ***p<0.001.

Figure 6 summarizes the average quantitative indices on all 50 testing datasets for original fast SPECT and DL-based denoised images. For all indices, DL-based denoising methods improve the image quality as compared to the original fast SPECT images. AttGAN-def obtains the best performance, followed by the AttGAN, cGAN, and Unet. At 1 s/prj fast SPECT images, the NMSE values are 0.0317 ± 0.007, 0.0337 ± 0.008, 0.0363 ± 0.011 and 0.0380 ± 0.013 for AttGAN-def, AttGAN, cGAN, and Unet, respectively, where AttGAN-def has significant difference with the other three DL methods. Similar results are obtained for the PSNR and SSIM values. The absolute errors of IR are 0.0435 ± 0.035, 0.0483 ± 0.046, 0.0498 ± 0.046 and 0.0632 ± 0.047 for AttGAN-def, AttGAN, cGAN and Unet on 1 s/prj fast SPECT images. For the absolute error of PDS, the AttGAN-def yields the lowest difference value among all denoising methods on all noise levels. The denoised images achieve better PDS performance, i.e., 1.60 ± 1.738, 2.36 ± 1.903, 2.76 ± 2.056 and 3.02 ± 2.428 for AttGAN-def, AttGAN, cGAN, and Unet on 1 s/prj fast SPECT images, as compared to the original fast SPECT images. The AttGAN-def has significant difference with the cGAN and Unet while not significant difference with AttGAN on 1 s/prj fast SPECT images. GAN methods outperform Unet in general.

#### 3.2.2. Bland–Altman plots

**FIGURE 7:**
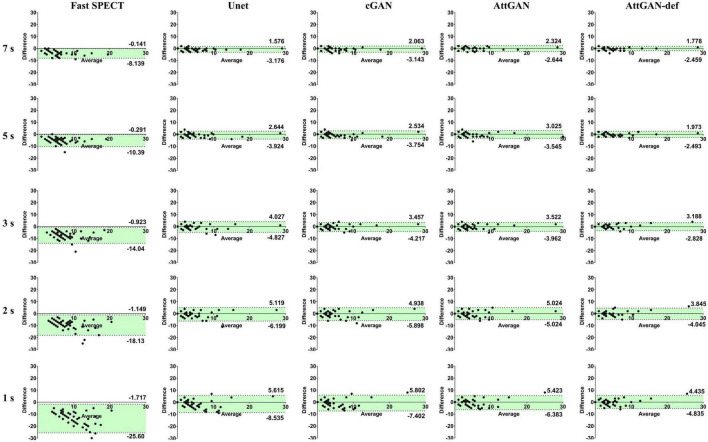
The Bland-Altman plots of PDS for fast SPECT different denoised images and acquisition times. The filtered FT SPECT images are used as reference.

The Bland–Altman plots of PDS for different denoised methods and fast SPECT images are shown in Figure 7. The dashed lines denote the 95% confidence interval (CI) of the PDS. For 1 s/prj fast SPECT, the AttGAN-def method shows the smallest variance (95% CI: −4.835, +4.435) compared to the reference filtered FT SPECT images, followed by the AttGAN (95% CI: −6.383, +5.423), cGAN (95% CI: −7.402, +5.802), and Unet (95% CI: −8.535, +5.615) methods.

#### 3.2.3. Joint histogram and linear regression analysis

**FIGURE 8:**
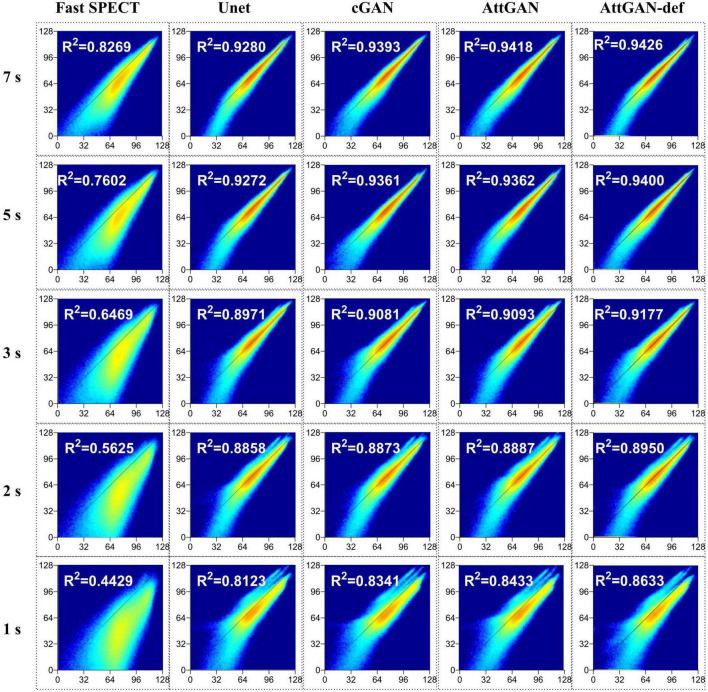
Joint histogram and linear regression analysis of fast SPECT and different denoised images in the 50 testing datasets for five shorter acquisition times. The filtered FT images are used as the reference.

The voxel-based joint histogram and linear regression analysis results are shown in Figure 8. Similar to other quantitative analysis, AttGAN-def obtains the best performance (R^2^= 0.8633), followed by the AttGAN (R^2^= 0.8433), cGAN (R^2^= 0.8341), and Unet (R^2^= 0.8123) on 1 s/prj fast SPECT denoised images.

## 4. Discussion

This study aims to assess the potential of attention-based DL network on fast MP-SPECT using clinical datasets. The attention module, inspired by non-local means, was proposed to enable the remote voxels to contribute to the local receptive filter during convolutional filtering (28). To the best of our knowledge, we are the first group to propose using an attention-based DL network for denoising the MP-SPECT images. For the quantitative performance comparison, we used filtered FT MP-SPECT images as the reference since there was no ground truth. Results consistently showed that the AttGAN denoised images had better quantitative accuracy from the difference images, polar plots, 17-segment plots, various physical indices, joint correlation histogram, linear regression and the clinical PDS analysis, as compared to conventional cGAN and Unet (Figures 4-8).

Our proposed networks were trained and tested for each dose-specific dataset, respectively, which were acquired on a CZT scanner. The denoising process is similar to an image-to-image translation task, and should be applicable to data acquired from other scanners or other denoised tasks, e.g., low dose SPECT imaging. The difference between low dose and fast SPECT is that the former will be more subject to patient motion with less radiation dose delivered to the patients. This is particularly important for the increasingly young patient population which has higher radiation risk than seniors (29). In our fast SPECT study, the reduction of acquisition time would be beneficial for patients with a sedation demand and patients with less compliance. The reduced acquisition time would further increase the patient throughput. Both low dose and fast SPECT are of clinical interest and can be potentially achieved using DL techniques (17).

Shiri et al. (17) suggested that denoising from half acquisition time per projection outperformed that from half number of projections for fast MP-SPECT by 2D ResNet. According to this reference, our fast MP-SPECT datasets were obtained by reducing the acquisition time per view. Compared with the existing literature, Ramon et al. (13) reported that CNN-based denoising on 1/2 dose level images could achieve image quality comparable to standard full dose images based on the TPD values. Aghakhan et al. (16) claimed that all denoised images on 1/2 dose level would be clinically acceptable by using 2D cGAN in the projection domain. Similarly, in our work, the mean value of the absolute difference for PDS, a similar index to TPD between FT and AttGAN-def denoised images on 1/2 acquisition time of FT is 0.66%. Although our 3D AttGAN results could not be directly compared with the previous studies due to the use of different networks, datasets, imaging protocols, and evaluation methods, the results are consistent.

In addition, some studies have shown that concatenating the gender, BMI, state (stress or rest), and scatter window images to the training dataset can improve the DL performance in the attenuation correction task (30). We first proposed to add patient defect information into the projections for training the AttGAN and showed promising results. Incorporating the defect information into the network structure or loss function could be further investigated but it is beyond the scope of this study. One should note that the defect information was extracted from the patients’ medical reports and SPECT images in this study, which may be subjected to potential image artifacts. Moreover, the actual defect information may not be able to be verified as other examination results of the patients, e.g., CT angiography, are not available in this study.

Ramon et al. (13) compared the performance between training on a specific low dose level and training on a collection of various low dose levels at the same time. Their preliminary results showed that a dose-specific network can be more accurate than a “one-size-fits-all” network. Liu et al. (31) proposed a denoising method using an image noise index calculated from the normalized standard deviation in the liver ROI for low dose PET images. The image noise index was embedded as a tunable parameter for training. Their results demonstrated that their denoising method achieved better denoising performance than the “one-size-fits-all” network, while it still could not outperform the dose-specific network. Thus, we trained our DL model using a dose-specific approach, i.e., separately for different image acquisition times, for the best denoising performance. More investigations are warranted for a more generalizable and efficient training strategy, i.e., transfer learning, data preprocessing and adjustment on the loss function for training based on all available data.

Limitations of this study include that the clinical-related evaluation is only conducted by PDS. A more comprehensive clinical analysis and a ROC study of defect detectability are needed to validate the proposed methodology. Another limitation is that our DL networks were trained based on a relatively small patient cohort, i.e., thirty-five patients. Training on a large number of patients would benefit the model performance with less susceptibility of overfitting, though our network’s loss function was validated to be converged. Gong et al. proposed to pre-train the DL network with simulation datasets and then fine-tuned it with a limited clinical dataset (32). Their results suggested that using simulation datasets with more realistic imaging conditions or with the use of a more accurate Monte Carlo simulation would generate a more robust pre-trained model.

## 5. Conclusion

In this work, we investigated the performance of AttGAN in denoising fast MP-SPECT images using clinical datasets. The proposed AttGAN provided superior denoising performance as compared to the conventional cGAN and Unet. Patient defect information could be useful parameter for further improving the AttGAN-based denoising performance.

## Data availability statement

The original contributions presented in this study are included in this article/supplementary material, further inquiries can be directed to the corresponding authors.

## Ethics statement

The studies involving human participants were reviewed and approved by Institutional Review Board of Taipei Veterans General Hospital (IRB number 2022-11-002CC). The written informed consent was waived due to the retrospective nature of this study.

## Author contributions

JS, T-HW, B-HY, and GM were the primary authors of the manuscript. JS was mainly responsible for data processing and analysis. YD was mainly responsible for data analysis and manuscript revision. C-YL was mainly responsible for data collection. T-HW, B-HY, and Y-HL were responsible for clinical interpretation. GM and T-HW were responsible for data interpretation and study integration. All authors contributed to the article and approved the submitted version.
